# Novel *CAD* gene mutations in a boy with developmental and epileptic encephalopathy 50 with dramatic response to uridine therapy: a case report and a review of the literature

**DOI:** 10.1186/s12887-024-04593-6

**Published:** 2024-03-07

**Authors:** Lifen Duan, Lei Ye, Runxiu Yin, Ying Sun, Wei Yu, Yi Zhang, Haiyan Zhong, Xinhua Bao, Xin Tian

**Affiliations:** 1 Epilepsy Center, Kunming Children’s Hospital (Children’s Hospital affiliated of Kunming Medical University), Kunming, 650031 China; 2 Department of Pediatrics, Peking University First Hospital, Beijing, 100034 China; 3 Department of Hematology, Kunming Children’s Hospital (Children’s Hospital affiliated of Kunming Medical University), Kunming, 650031 China

**Keywords:** *CAD* gene, Mutation, DEE-50, Uridine, Epilepticus

## Abstract

**Background:**

Developmental and epileptic encephalopathy-50 (DEE-50) is a rare clinical condition believed to be caused by a mutation in the *CAD* gene and is associated with a bleak prognosis. *CAD*-related diseases have a wide range of clinical manifestations and other symptoms that may be easily overlooked. Like other rare diseases, the clinical manifestations and the treatment of DEE-50 necessitate further investigation.

**Case presentation:**

A 1-year-old male patient presented with developmental delay, seizures, and anaemia at 3 months of age. He further developed refractory status epilepticus (SE), rapid deterioration of cognitive and motor function, and even became comatose at 5 months of age. Whole-exome sequencing of trios (WES-trios) revealed a compound heterozygous variant in the *CAD* gene, with one locus inherited from his father (c.1252C>T: p.Q418* nonsense mutation) and one from his mother (c.6628G>A: p.G2210S, missense mutation). This compound heterozygous CAD variant was unreported in the Human Gene Mutation Database. After uridine treatment, his cognitive faculties dramatically improved and he remained seizure-free.

Forty two cases with *CAD* gene mutation reported in the literatures were reviewed. Among them, 90% had onset before 3 years of age, with average of 1.6±1.8 years old. The average age of diagnosis was 7.7 ± 10 years. The mortality rate was approximately 9.5%, with all reported deaths occurring in patients without uridine treatment. The clinical entity could be improved dramatically when the patient treated with uridine.

**Conclusions:**

We present a boy with DEE 50 caused by novel* CAD* gene mutations and reviewed the clinical features of 42 patients reported previously. DEE 50 has early onset, refractory seizures, even status epilepticus leading to death, with favorable response to treatment with oral uridine. Early uridine treatment is recommended if CAD defect is suspected or genetically diagnosed. This study enhances the knowledge of DEE 50 and expands the spectrum of *CAD* gene mutations.

## Background

The primary objective in the field of genetic metabolic diseases is to diagnose these conditions precisely, enabling the formulation of targeted treatment plans. For example, pyridoxine-dependent epilepsy, biotinidase deficiency, glucose transporter 1 deficiency, and cerebral creatine deficiency syndrome are clinical entities that can be well mitigated by targeted treatment following a precise diagnosis. A specific example is uridine-responsive epileptic encephalopathy, in which patients respond to uridine treatment after the diagnosis is established [1].

The first report of a *CAD* gene mutation in epileptic encephalopathy patients emerged in 2015 [1]. Despite the potentially fatal progression of this disease, it stands as one of the few genetic metabolic disorders with a significantly effective specific treatment. The *CAD* gene encodes three functional enzymes (carbamoyl phosphate synthetase, aspartate transcarbamoylase, and dihydroorotase) in the *de novo* biosynthetic pathway of pyrimidines [2–4]. Subsequently, mutations affecting the *CAD* gene can lead to defects in pyrimidine metabolism, with the primary clinical manifestation being DEE. In addition, other clinical phenotypes were described, such as developmental delay/regression, anaemia with poikilocytosis, and ataxia.

To date, 42 cases of *CAD* variants have been reported [2, 5–14]. In this study, we summarise the clinical manifestations of such variants and present previously overlooked clinical phenotypes, such as dysphagia, hypotonia, and epilepsy related to fever sensitivity. In addition, we also present detailed a case with DEE 50 caused by novel *CAD* gene mutation, who was treated at the Epilepsy Center of Kunming Children’s Hospital, Yunnan, China.

## Case presentation

A case of a Chinese male patient with developmental delay and regression, refractory epilepsy, and anaemia is presented in this study. Relevant clinical information, including disease onset, clinical manifestations, family history, results of ancillary tests, and outcomes of uridine treatment, were collected and analysed. Genomic DNA was extracted from the peripheral leukocytes of the patient, his parents, and his sister. The study protocol was in accordance with the Declaration of Helsinki, and written informed consent was obtained from the patient’s guardian. Written approval was obtained from the Ethics Committee of Kunming Children’s Hospital (2021-03-055-K01).

We searched and reviewed the relevant literature on PubMed (https://pubmed.ncbi.nlm.nih.gov/) based on the keywords “CAD,” “carbamoyl-phosphate synthetase, aspartate transcarbamoylase, and dihydroorotase,” “developmental and epileptic encephalopathy-50,” and “uridine” as of 31 December 2022. We found 12 publications [2, 5–14], each presenting cases with their corresponding general information, clinical manifestations, electroencephalography (EEG) and brain magnetic resonance imaging (MRI) findings, gene mutations, and treatment and prognosis.

### Clinical information

This patient was a 1-year-old boy, born via normal vaginal delivery at full term after an uncomplicated pregnancy, with a birth weight of 2.5 kg and no specific family history. At 3 months of age, he presented with anorexia, vomiting, abdominal distension, anaemia, and spasmodic seizures (1–2 times daily). Initial physical assessment revealed that the patient was developmentally delayed, evident by his inability to hold up his head steadily, poor smooth pursuit, dull eyes, and no active laughing. Physical examination revealed anaemic appearance and hypotonia. The patient was started on anti-seizure treatment with sodium valproate (30 mg/kg), after which he remained seizure-free for the following 2 months. At 5 months, the patient presented with recurrent episodes (more than 20 times per day), which were characteristic of migrating focal epilepsy and refractory status epilepticus (SE); he also exhibited rapid deterioration of cognitive and motor function, eventually entering a comatose state. The Glasgow Coma Scale (GCS) was 8 (minimum score 3 and maximum score 15, and a score <8 rated were as took as coma). Dysphagia developed rapidly and necessitated feeding via a nasogastric tube.

The patient was treated with an infusion of suspended erythrocytes and five anti-seizure medications (ASMs): sodium valproate, oxcarbazepine, topiramate, phenobarbital, and midazolam consecutively.However, the seizures couldn’t be controlled with. Besides having more than 10 times focal seizures daily, the patient also suffered from gastrointestinal bleeding, dysphagia, and severe pneumonia. To address this, the patient was treated with aggressive resuscitation therapy for 2 months, which included maintaining the patient jejunitas and on gastric acid suppression therapy, haemostasis, anti-microbial therapy, and high-flow ventilation. Following the gradual discontinuation of treatment with midazolam and phenobarbital, the patient kept experiencing seizures even on maintenance treatment with sodium valproate (30 mg/kg·d), oxcarbazepine (30 mg/kg·d), and topiramate (6 mg/kg·d). The patient still had impaired consciousness (GCS: 9), no cognitive function recovery, and limited dietary intake (only powdered milk).

**Fig. 1:**
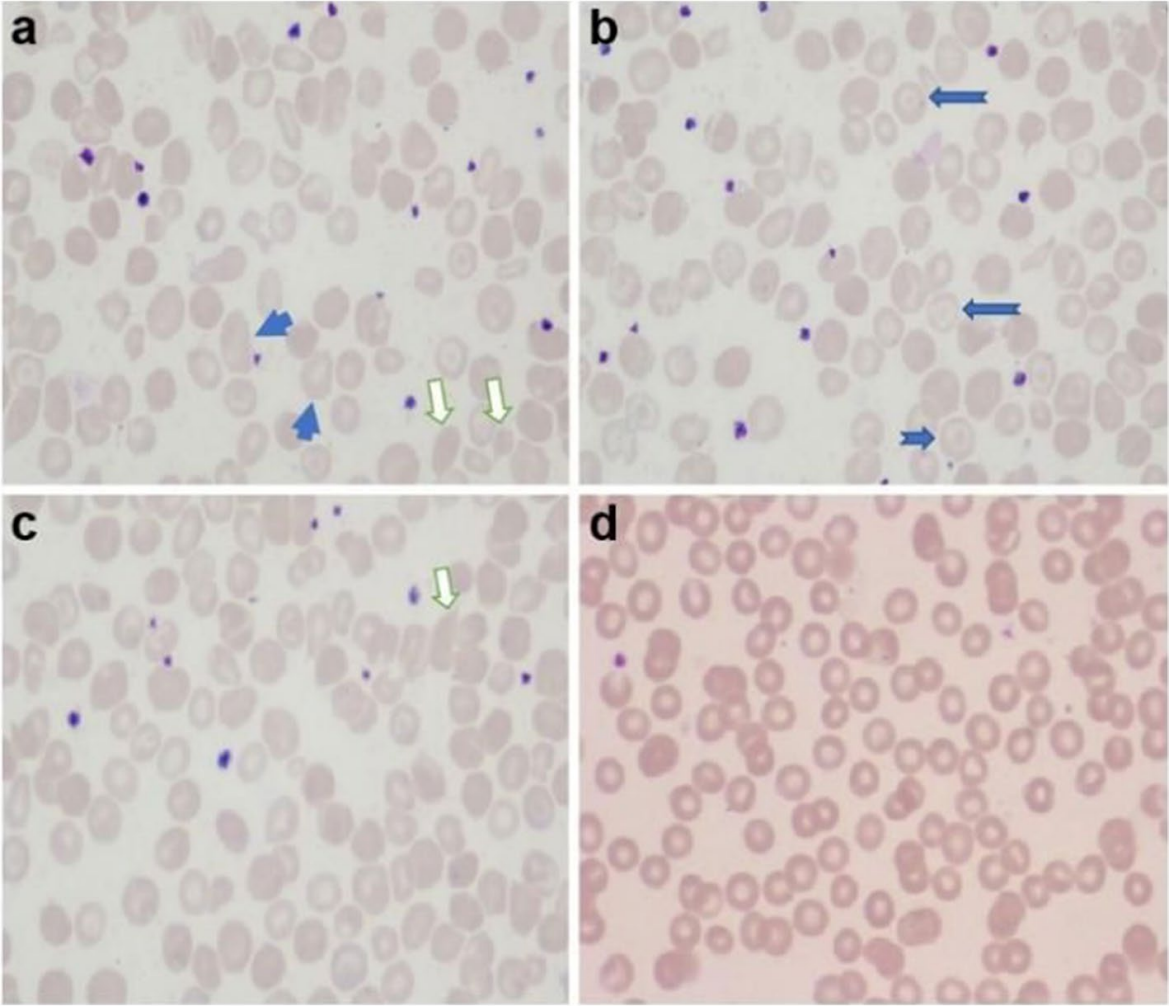
Erythrocyte smear. **a**–**c** Before treatment. The mature erythrocytes are slightly variable in size, and the lightly stained areas in the centre of some erythrocytes are enlarged, with a few target erythrocytes, teardrop cells, and acanthocytes (  target erythrocytes,  teardrop cells,  acanthocytes). **d** After treatment. Normal erythrocyte morphology and disappearance of abnormal erythrocytes

**Fig. 2:**
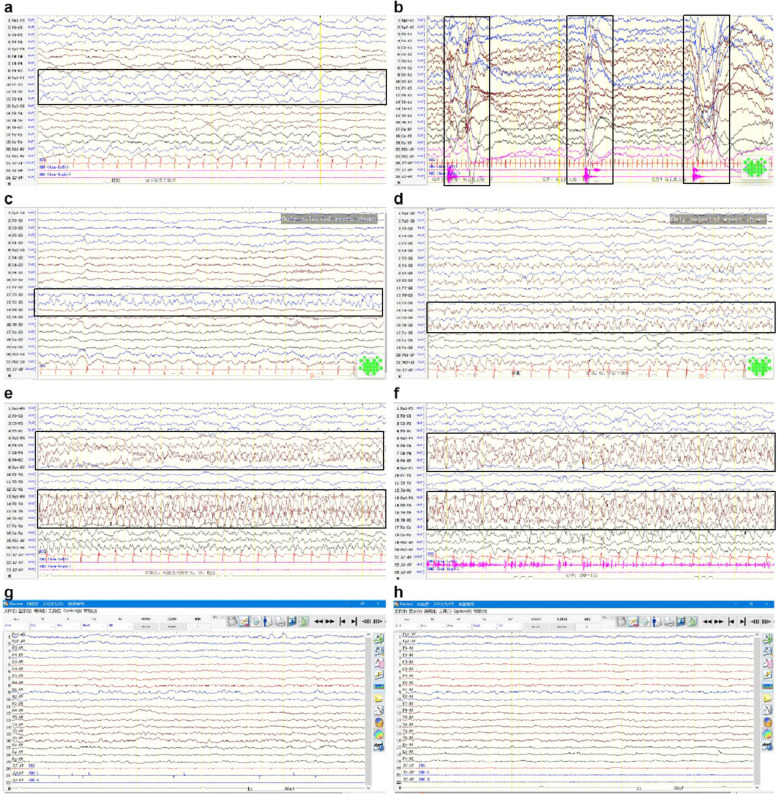
Electroencephalography (EEG) findings. **a**–**b** EEG at 3 months: **a** Continuous bursts of sharp and slow waves in the left temporal region, especially in the anterior inferior temporal region. b. Series of spastic seizures. **c**–**f** EEG at 6 months: **c** Bursts of spike and sharp waves in the left posterior temporal region; bursts of fast waves in the bilateral parietal, occipital, and middle posterior temporal regions; **d**–**f**. Focal seizures initiated in the right temporal region. **g**–**h** EEG at 1 year: Follow-up EEG after treatment showed normal results

**Fig. 3:**
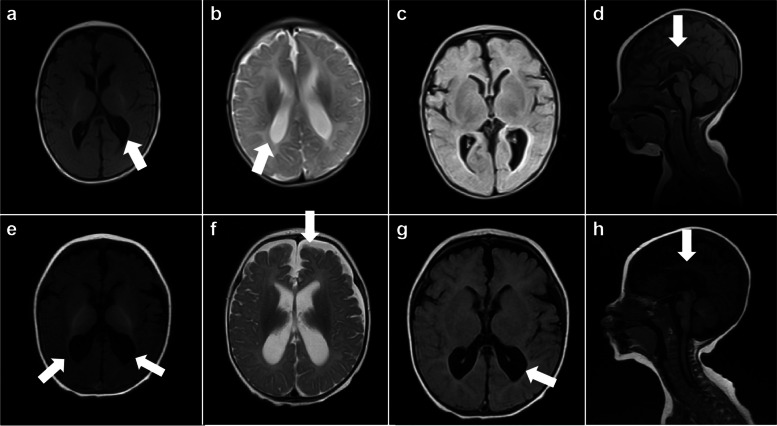
MRI findings. **a**–**d** (a–c: axis view, d: sagittal view) (at 3 months) Slightly enlarged posterior horn of the bilateral ventricles and hypoplasia of the corpus callosum(white arrow). **e**–**h** (e–g: axis view, h: sagittal view) (at 6 months) Enlargement of the bilateral ventricles compared with the previous ones, widening of the extracerebral frontal space, hypoplasia of the corpus callosum, and delayed myelination(white arrow)

Ancillary tests were performed. Routine blood tests suggested microcytic hypochromic anaemia (haemoglobin level: 66 g/L, erythrocyte count: 3.42×10^12^/L, haematocrit: 22%, mean erythrocyte volume: 62 f l, and mean erythrocyte haemoglobin content: 18 pg). The erythrocyte smear revealed that the mature erythrocytes were slightly variable in size, and the lightly stained areas in the centre of some erythrocytes were enlarged, with a few target erythrocytes, teardrop cells, and acanthocytes (Fig. 1a–c). The patient had normal values for blood biochemistry, thyroid function tests, ceruloplasmin, folic acid, and vitamin B12 levels. The analysis of amino acids and acylcarnitines spectrum by MSMS in blood and the profile of organic acids in urine by GCMS were normal. Multifocal abnormal discharges and spastic seizures were observed on EEG at 3 months (Fig. 2a, b), compared with multifocal discharges, focal electrical seizures, and clinical seizures observed at 6 months (Fig. 2c–f). Cranial MRI at 3 months revealed enlargement of the posterior horn of bilateral ventricles and hypoplasia of the corpus callosum (Fig. 3a–d). Follow-up MRI at 6 months revealed further enlargement of the ventricles and widening of the extracerebral frontal space and hypoplasia of the corpus callosum as before (Fig. 3e–h).

### Genetic test

We took a Trio-WES strategy to identify the causal variants from the family trios. In brief, genomic DNA was extracted, hybridized and enriched.Firstly, 1μg genomic DNA was extracted from 200μL peripheral blood, using a Qiagen DNA Blood Midi/Mini kit (Qiagen GmbH, Hilden, Germany) following the manufacturer’s protocol. 50 nanogram DNA was interrupted to 200bp around by fragmentation enzymes. The DNA fragments were then end repaired, and the 3’end was added one A base. Secondly, the DNA fragments were ligated with barcoded sequencing adaptors, and fragments about 320bp were collected by XP beads. After PCR amplification, the DNA fragments were hybridized and captured by Berry’s NanoWES Human Exome V2.0(Berry Genomics,Beijing,China) according to the manufacturer’s Protocol. The hybrid products were eluted and collected, and then subjected to PCR amplification and the purification.Next, the libraries were quantified by qPCR and size distribution were determined using an Agilent Bioanalyzer 2100 (Agilent Technologies, Santa Clara, CA, USA).Finally, Novaseq6000 platform (Illumina, San Diego, USA), with 150 bp pair-end sequencing mode, was used for sequencing the genomic DNA of the family. Raw image files were processed using CASAVA v1.82 for base calling and generating raw data.

The sequencing reads were aligned to the human reference genome (hg38/GRCh38) using Burrows–Wheeler Aligner tool and PCR duplicates were removed by using Picard v1.57(http://picard.sourceforge.net/). Verita Trekker^®^ Variants Detection System by Berry Genomics and the third-party software GATK (https://software.broadinstitute.org/gatk/) were employed for variant calling. Variant annotation and interpretation were conducted by ANNOVARand the Enliven^®^ Variants Annotation Interpretation System authorized by Berry Genomics.

We used SWISS-MODEL to simulate the prominent amino acid and conformational changes in the influenced polypeptide. Sequence information was downloaded from NCBI database and comparative analysis was conducted by UGENE software. Previously used to predict functional domains from NCBI and draw graphs using IBS.

**Fig. 4:**
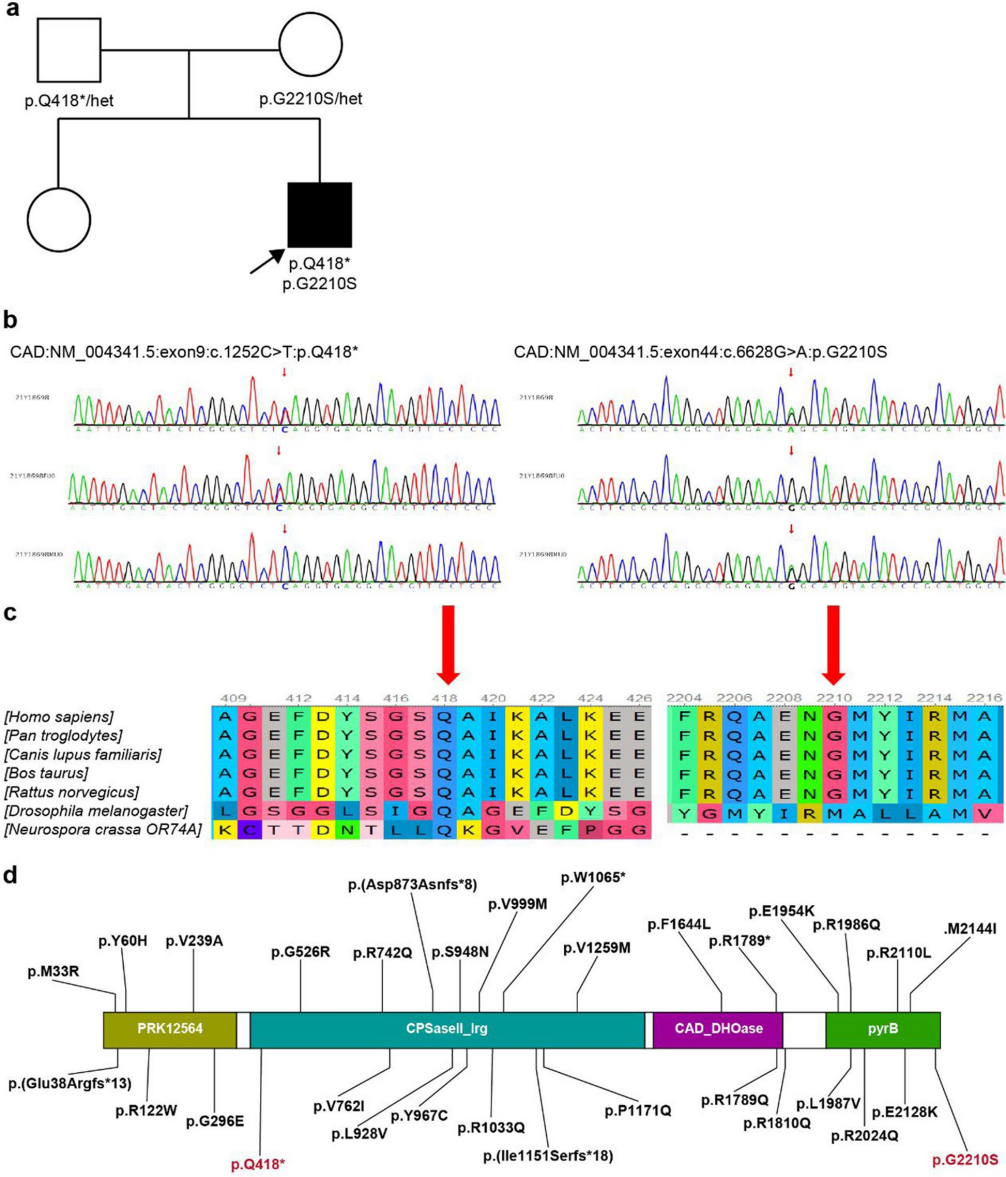
Functional domains. **a** Pedigree chart (black arrows indicate probands). **b** Sanger sequencing of CAD variants. **c** Conservation of the variants found in this study. **d** Schematic view of the CAD variants based on NM_004341.5. CAD contains four subdomains, a PRK12564 domain (grass green), a CPSasell_lrg (blue-green), a CAD_DH0ase (purple) and a PyrB (green). Red fonts indicate variants reported in this study

**Fig. 5:**
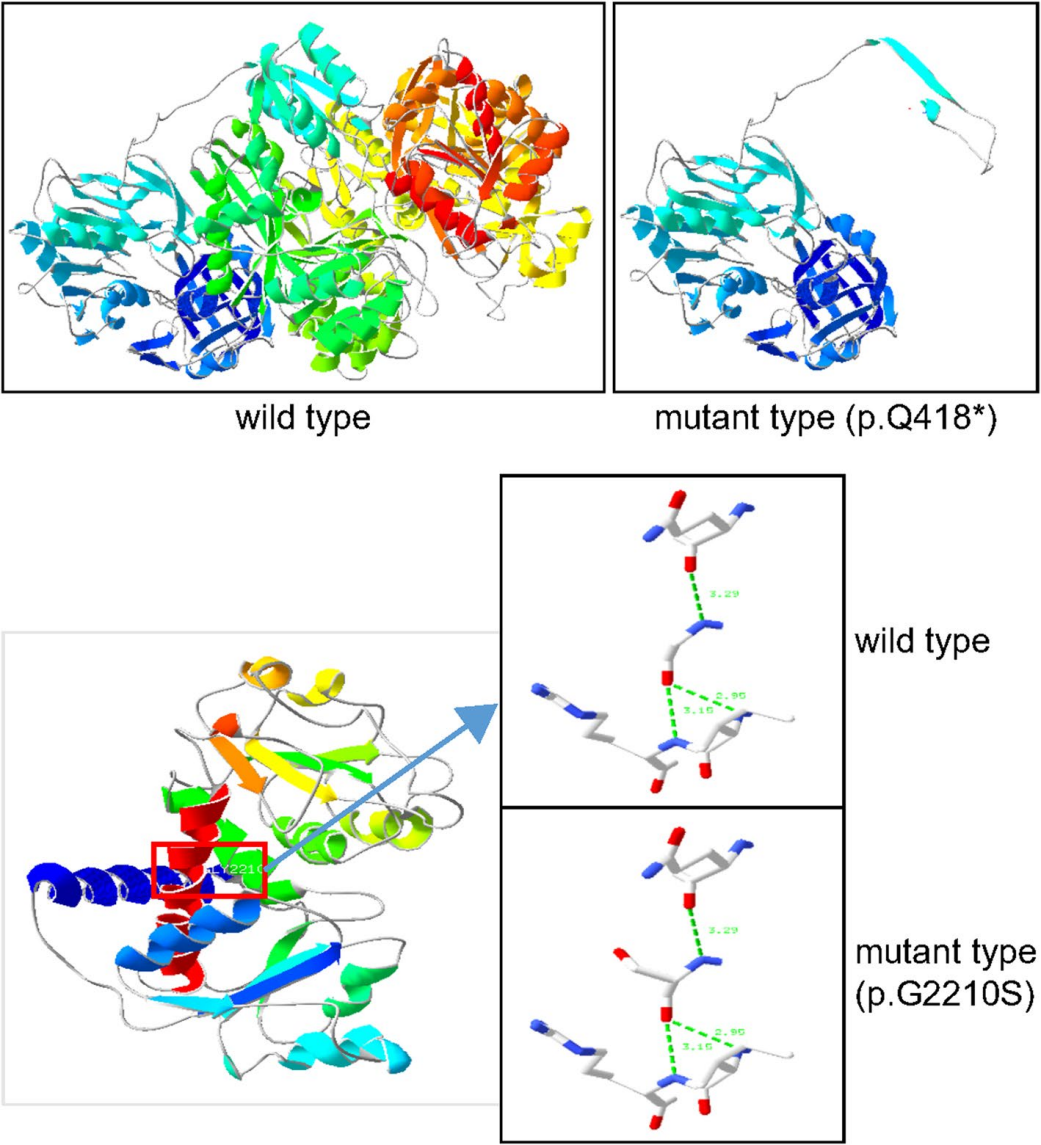
Molecular modelling of the missense variant. p.Q418*: significant change in protein structure after truncation mutation. G2210S: no change in the linking hydrogen bond but change in conformation

The gene mutational test revealed compound heterozygous variants (c.1252C>T, p.Q418*; c.6628G>A, p.G2210S) in the *CAD* gene, of which were inherited from the father and the mother respectively. The patient’s sister, however, did not carry the mutation (Fig. 4a–b). According to the guidelines of the American College of Medical Genetics and Genomics (ACMG), the c.1252C>T variant is considered to be pathogenic (PVS1+PM3+ PM2_supporting), and the c.6628G>A variant is considered to be likely pathogenic (PP3_Strong+ PM2_supporting+ PP2). Neither of these two variants has been reported previously. Both loci were relatively conserved across multiple species (Fig. 4c), suggesting that they may be essential in maintaining protein stability and function. The distribution of *CAD* gene mutation was shown in Fig. 4d. The three-dimensional prediction revealed significant protein structure changes caused by the mutations, which may suggest an overall effect on the conformation and activity of the protein (Fig. 5).

### Treatment and follow-up

Genetic testing performed at 7 months confirmed the diagnosis of DEE-50 due to *CAD* mutations. Uridine treatment was initiated immediately, gradually increasing from 50 (mg/kg·d) to 100 (mg/kg·d) divided in three doses. One week after initiating treatment, the seizures were controlled completely. As well, both the cognitive function and the state of consciousness improved significantly. At the 5-month follow-up appointment, the patient was 1-year old and seizure-free, with normal EEG findings (Fig. 2g, h), and has made significant progress in overall development.He could feed himself, makes eye contact with the family, laughs, babbles, holds his head up, rolls over, grasps objects on his initiative, sits alone, and walks with support. However, his Griffiths scale still lagged behind normal children (Table 1). The anaemia also resolved, and the anomalous erythrocytes disappeared (Fig. 1d). Treatment with topiramate was eventually discontinued, and the patient continued treatment with sodium valproate (30 mg/kg·d) and oxcarbazepine (30 mg/kg·d), with a plan to taper and to terminate the ASM if there was no recurrence.

**Table 1:** Griffiths scale

**subscales**	Locomotor	Personal-social	hearing and language	Hand-eye coordination	Performance
**raw scores**	10.3	9.3	7.2	7.5	8.6
**functional age**	9.5-10m	8-8.5m	6.5-7m	7.5-8m	9m
**percentage**	20-25%	7.5-10%	25-30%	5-7.5%	12.5-15%

### Literature review

After searching the literature, 42 reported cases were included in this study (Table 2). Of them, the sex of 30 cases was provided, with 17 male and 13 female. The mean age of onset was 1.6 ± 1.8 years (early childhood), with 90% of onset starting before the age of 3 years and as early as on the second day of life. Among them, three cases had the onset in the neonatal period, 12 cases between 1 and 12 months, no adult onset was reported. The age at diagnosis was 7.7 ± 10 years on average (school age) and ranged from 9 months to 43 years with a median of 5.5 years, only two cases were diagnosed in adult. Five patients had consanguineous parents, and 12 had a reported family history. The clinical features of the 42 patients included developmental delay or regression (95%), epilepsy (73%), anaemia (71%), extrapyramidal symptoms (33%), dysphagia (23%), hypotonia (14%), and strabismus (7%) (Table 2). Moreover, 31 patients had seizures, 76% of whom had the onset before the age of 2 with the mean age of 1.8 ± 1.7 months and a median of 1.5 years. The mean number of ASMs used was 3.72 ± 1.36. Twenty cases had drug-refractory epilepsy, fourteen had SE, and one had a history of temporal lobectomy and ventriculoperitoneal shunt surgery. Within 31 cases with drug-refractory seizures, 4 had heat-sensitive epilepsy (13%). A total of 18 patients received uridine treatment. This included seven patients treated with uridine (100 mg/kg·d), eight treated with uridine monophosphate (141 ± 36 mg/kg·d), and three treated with uridine triacetate (TAU) (110 ± 14 mg/kg·d). No fatalities were recorded after the treatment. Moreover, the calculated mean of ASMs following uridine treatment was 2.1 ± 1.3. In addition, of the patients with persistent SE, seven reached seizure freedom after treatment, and four had residual seizures but no persistent SE. Interestingly, three SE patients were seizure-free on day two of treatment. As for improvements in EEG findings after treatment, seven cases had significant improvement, while two did not. In addition, 15 cases had their anaemia corrected at approximately 2.3 months of treatment. Four patients died (9.5%, all of whom did not receive uridine treatment), with a mean age at death of 3.8 ± 1.2 years and a median of 4 years.

**Table 2:** Clinical information of patients reported in the literature

			Numbers	Percentage
**Total cases**			42	
**Clinical manifestation**				
**Developmental disorder**			40/42	95
	Developmental delay		34/40	85
		Autism spectrum disorder	4/40	10
		Minimum state of consciousness	4/40	10
	Developmental regression		6/40	15
**Epilepsy**			31/42	73
	Drug-refractory seizure		20/31	64
	SE		14/31	45
	Fever-induced or exacerbated seizure		4/31	13
	Seizure types		24/31	77
		Focal seizure	10/24	41
		GTCS	9/24	37
		Myoclonic seizure	2/24	8
		Tonic seizure	1/24	4
		Atonic seizure	1/24	4
		Absence epilepsy	1/24	4
**Anaemia**			30/42	71
	Anisocytosis		10/30	33
	Normal cell anaemia		5/30	16
	Small cell anaemia		2/30	6
	Large cell anaemia		1/30	3
**Extrapyramidal symptoms**			19/42	45
	Ataxia		14/19	73
	Tremor		7/19	36
	Gait abnormalities		5/19	26
**Dysphagia**			10/42	23
**Hypotonia**			6/42	14
**Supplementary examination**				
**MRI**			24/42	57
	Brain atrophy		11/24	73
		Whole brain atrophy	7/24	29
		Cerebellar atrophy	4/24	16
	Hydrocephalus		2/24	8
	Delayed myelination		2/24	8
	Agenesis of corpus callosum		1/24	4
	Enlargement of lateral ventricle		1/24	4
	Widening of extensive extracerebral space		1/24	4
**EEG**			11/42	26
	Background	Normal	6/11	54
		Slow wave	5/11	45
	Interictal Phase	Multifocal discharges	6/11	54
		Focal discharges	2/11	18
	Ictal phase	Focal seizures	4/11	36
		Absence seizure	1/11	9
		Myoclonic seizure	1/11	9
***CAD* mutations**			42/(80 site)	100
	Missense mutations		63/80	78
	Nonsense mutations		12/80	15
	Splice site mutations		5/80	6
**Uridine treatment**			18/42	42
**Development**	Significant progress		16/18	88
	Not obvious progress		2/18	11
**Epilepsy**	Significantly reduced		15/18	83
		Seizure-free status	11/18	73
	No improvement		3/18	16
		Uridine monophosphate	2/18	11
		Received TAU	1/18	5
	SE	Seizure-free	7/18	38
		Residual seizures	4/18	22
**Anaemia**	Correction		15/18	83

*Abbreviations*: *SE* Status epilepticus, *MRI* Magnetic resonance imaging, *EEG* electroencephalography, *GTCS* Generalized tonic-clonic seizures, *TAU* uridine triacetate

## Discussion

Mutations in the *CAD* gene are thought to be primarily responsible for the development of DEE-50, an inherited autosomal recessive disorder known for its progressiveness and poor prognosis [5]. The main clinical manifestations of this disorder are drug-refractory epilepsy, psychomotor retardation/loss of skills, anaemia, and progressive brain atrophy. Some patients can present only developmental delay and not always experience seizures. In general, developmental delay seems to be present in the majority of cases [12]. Luckily, DEE50 can be effectively treated with uridine [6, 12].

In this study, we present a case with novel *CAD* mutation, and reviewed the clinical features of 42 reported cases. The major clinical phenotypes of these patients include developmental delay or regression, epilepsy, anaemia, extrapyramidal symptoms, dysphagia, hypotonia, and strabismus. The patient we described in this study presented nearly all these symptoms, except extrapyramidal symptoms and strabismus. Some manifestations, such as extrapyramidal symptoms, dysphagia, and hypotonia, have been underappreciated in previous reports. This study, however, acknowledges this and highlights the high incidence of extrapyramidal symptoms in approximately one-third of the cases, marking it as an essential phenotype of the disease as it can be reflective of cerebellar atrophy [12]. Further studies should focus on other overlooked symptoms, such as dysphagia and hypotonia.

CAD deficiency may manifest early in the neonatal period, with a mean age of onset was 1.6 ± 1.8 years (early childhood), with most cases presenting before the age of 3 years. However, the average age of diagnosis was 7.7 ± 10 years (school age), indicating an approximately 6-year lag between onset and diagnosis. This underscores a significant delay between diagnosis and treatment initiation. In contrast, the patient in our study, diagnosed via genetic testing, was considerably younger, at 7 months.

The disease has a high mortality rate, and the deaths reported so far have been in patients who have not received uridine,thus following the natural fatal course of the disease. Most patients exhibit a severe clinical phenotype, manifesting as persistent SE and developmental regression, and can even reach a reduced state of consciousness. The patient in this study exhibited severe manifestations, such as persistent refractory SE, and entered a comatose state. In addition, he also developed serious complications such as gastrointestinal bleeding and severe pneumonia, which led to 2 months of hospitalisation.

Based on this case and literature review, the epileptic phenotypes associated with *CAD* mutations occur before the age of 2 years in most cases. The predominant presentation picture is focal seizures. One of the reviewed cases from the literature had a history of temporal lobectomy. Preoperative evaluation should take carefully in order to avoid surgical treatment on the patients with treatable metabolic diseases. Drug-resistant epilepsy was prevalent in patients with *CAD* mutation-related epilepsy, with nearly half of the patients suffering from persistent SE and four having heat-induced or exacerbated seizures. Moreover, heat-sensitive epilepsy may also be an early warning sign of this disease. As reported in the results section, anisocytosis was present in only one-third of the anaemia cases. Anisocytosis with or without anaemia in patients with refractory epilepsy may also be an essential clue to suspect *CAD* defects. The pathophysiology behind the hematopoietic dysfunction seen in such patients may be explained by the lack of pyrimidine-dependent nucleotide-lipid cofactors required for erythrocyte membrane synthesis [2, 12]. Mild anaemia can be corrected by nutrition supplementation, while anisocytosis can only be corrected by oral uridine treatment.

The *CAD* gene is located on chromosome 2p23.3 and contains 44 exons, encoding a highly conserved multifunctional enzyme complex containing 2,225 amino acids [10]. The CAD enzyme complex is essential in the *de novo* synthesis pathway of pyrimidines where it plays a role as a key enzyme in the first three steps of the pathway that results in uridine-5-monophosphate (UMP) synthesis [12]. UMP is the substrate for all cellular pyrimidines and is therefore essential for RNA and DNA synthesis.Therefore, *CAD* mutations are expected to lead to impaired pyrimidine nucleotide synthesis. This is reflected in a wide range of molecular/cellular dysfunctions in organismal nucleic acids, protein glycosylation, lipid metabolism, polysaccharide biosynthesis, and signal transduction and proliferation [5]. Pyrimidine metabolism disorders may also interfere with the neuronal differentiation process, which can impair axon and dendrite formation and lead to neuronal migration disorders [3]. In this case, mutations were predicted to result in altered tertiary protein configuration or deteriorated protein function. *CAD* expression is the highest in the cerebrum, cerebellum, kidney, and peripheral nervous system [13]. In literature review, the patients were mainly involved in the central system. The involvement of other systems except the blood system was relatively rare, and peripheral nerve involvement was not reported.

Although *CAD* defects lead to impaired pyrimidine biosynthesis and in turn impaired glycosylation as described above, routine metabolites such as urinary and serum pyrimidines, hypoxanthine, xanthine, and transferrin isoelectric focusing are often at normal levels [2]. Moreover, urine orotic acid levels before uridine supplementation are usually within the normal range. Hence, such metabolites are not suitable to act as biomarkers for predicting the presence of an underlying *CAD* mutation [7]. Although some patients show background slowing on EEG dominated by focal and multifocal discharges, specific EEG changes cannot be expected. Our study demonstrated that one-third of the reviewed cases had MRI abnormalities, with delayed myelination and brain atrophy being the main manifestations in infants and older children, respectively. The cerebral and/or cerebellar atrophy gradually progressed after 3.5 years of age. Based on these observations, it is evident that a definitive diagnosis of CAD deficiency requires genetic testing if we consider the lack of reliable biomarkers and absence of specific changes on EEG and MRI (although some findings may be suggestive in some cases) [12].

Exogenous uridine is thought to overcome the loss of CAD function, bypassing the defect in the *de novo* pyrimidine synthesis pathway and its downstream pathways. Three exogenous forms of uridines are currently available clinically: uridine, UMP, and TAU. TAU is a uridine precursor that is converted to uridine *in vivo* and is an FDA (Food and Drug Administration)-approved drug for treating of hereditary orotic aciduria [5]. TAU is four to six times more bioavailable than uridine, requiring a lower dose to achieve the therapeutic range. TAU protein is absorbed in the gastrointestinal tract and readily converted by esterases in the intestinal epithelium to free uridine and acetate. However, two considerations of treatment with TAU are that it is more expensive than the other two drugs and the possibility that it can rarely cause side effects such as mild nausea, vomiting, or diarrhoea [5]. Some studies report that treatment with TAU does not seem superior to treatment with uridine or UMP. Rymen D reported that the frequency of epilepsy didn’t reduce in two of three cases treated with uridine monophosphate and TAU [12], compared to the case presented in this study, which showed significant clinical improvement. Whether uridine is superior to the other two drugs remains to be further explored in future studies. Furthermore, studies performed on Drosophila *CAD* mutants showed that the organisms could not survive on growth media devoid of pyrimidines. They could, however, survive when the media was supplemented with pyrimidines, although they would exhibit features such as small wings, abnormal structural features, and female sterility [15]. Hence, the presence of uridine was able to prevent the lethal natural course of CAD deficiency but not all other phenotypic defects, suggesting that certain tissues may be preferentially dependent on the *de novo* synthesis pathway compared with other tissues, which may be dependent on the salvage pathway of pyrimidine synthesis. This is consistent with the findings of some in vitro functional studies performed on fibroblasts derived from patients with CAD deficiency, which showed reduced concentrations of pyrimidine nucleotides. Upon supplementation with uridine in the culture medium, pyrimidine levels were soon corrected, suggesting replenishment had occurred. This is in line with the mechanism of the pyrimidine salvage pathway, which allows pyrimidine nucleotides to be replenished and the biochemical processes that are dependent on pyrimidine metabolites to be restored. This is possible due to the ability of cells to take up and phosphorylate exogenous uridine, hence allowing UMP to be synthesised through the salvage pathway [12].

The successful utilisation of uridine in treating epileptic encephalopathy secondary to *CAD* mutations is further evidence of the importance of implementing genetic technology in precision medicine [5]. Early infantile epileptic encephalopathy-50 is one of the rare genetic disorders that is treatable with promising results, arguing for the importance and value of precision medicine [6]. Most patients reviewed in this study showed dramatic improvement in clinical symptoms, including drug-refractory epilepsy and persistent SE, after uridine treatment [12]. Uridine treatment may enable patients to discontinue the unwarranted use of antiepileptic drugs and to avoid their potential side effects. Among the cases reviewed in this study, significant cognitive developmental progress was documented in 16 cases after initiation of uridine treatment, with only three showing no significant progress. Additionally, no fatalities or serious adverse effects were reported in the treated cases, indicating the safety of uridine supplementation therapy in patients with CAD deficiency. Considering the fatal natural course of this entity and the aforementioned safety profile of the treatment, uridine treatment should be attempted in patients who show signs of early developmental delay and refractory epilepsy and additional symptoms such as anaemia and anisocytosis even before the definitive genetic diagnosis is confirmed and that CAD should be considered to be included in neonatal genetic screening [12]. Ultimately, this targeted therapy is promising for the ever-evolving medical field because of its reassuring safety profile.

## Conclusions

This study presented a case of novel CAD-mutation related DEE-50 and with detailed fellow up from onset to diagnosis to treatment. The results from the genetic testing performed on the patient were added to the mutational spectrum of CAD. In addition, the cilical characteristic of 42 cases with DEE50 were reviewed. In summary, CAD deficiency is a rare disease with infantile onset and a potentially fatal natural course. The mortality rate is approximately 9.5%, with all reported deaths occurring in those without uridine treatment. Moreover, the age of diagnosis significantly lags. The clinical manifestations include developmental delay/regression, refractory epilepsy, anaemia, extrapyramidal symptoms, dysphagia, and hypotonia. There are no specific biomarkers or predictable changes on EEG, and although brain atrophy may be seen as a manifestation on late MRI, it is also not a specific finding. A definitive diagnosis can be achieved by *CAD* mutation analysis, and uridine treatment has dramatic efficacy. Because uridine has a good safety profile, tentative uridine treatment may be recommended for those who present with the above-mentioned clinical manifestations, and it is advised to genetically screen for the *CAD* gene in neonates for early diagnosis and prevention.

## Data Availability

The datasets generated and/or analysed during the current study are available from the corresponding author on reasonable request.

## Abbreviations

DEE: Developmental and epileptic encephalopathy
EEG: Electroencephalography
MRI: Magnetic resonance imaging
TAU: Uridine triacetate
SE: Status epilepticus
